# Nurses in the Sick Room

**Published:** 1892-10

**Authors:** S. Weir Mitchell


### NURSES IN THE SICK ROOM.

I confess to being a little shy of the new nurse graduate. And now I shall be personal, and say that here character begins to show. A nurse may know more than enough and disastrously fail for want of some moral qualities ; and this is a shameful thing, to fail where you have full control. Failure of the head one may pardon ; failure of the heart—ah, that is quite another thing. I think real, wholesome sweetness of temper a fine thing in nursing. If it come out of a wise and large charity as to the suffering and nervous, it is of utmost value. Never worry or be worried If a patient will not do as she should, set it all aside for the time ; refer it to the doctor; above all, don't get the patient out of temper.

My own nursing demands the largest goodness of temper—not to be merely amiable. Think a little what goodness of temper means. If it be united with firmness and tact, it becomes of highest use. I like to say that one of Miss Swain's nurses of late let me see a fine example of weeks' of utter devotion, tact, firmness of temper, with the most profound interest in her case and a resolute will to win. Now that is a matter of moment—the interest in a case. I hate a nurse to say, "Oh, I can't nurse this or that ; it don't interest me." I like her to steadily want to help people, and to use all her energies persistently for this purpose.

I like her to share my intense desire to make people well—my profound dislike to being defeated. I remember asking General Sheridan once if he had ever thought out the cause of his constantly victorious career. He said yes; that he believed what success he had had to have been due to his "so hating to be licked." It must be your life business not to be too easily beaten.

I also advise you not to expect or desire to make a friend of a patient, or to become intimate. I remember when young in my work my father saying to me: "Let your patient make a friend of you. Do not always try to make friends of them. Patients get notions and leave you, and then you are hurt by their leaving you." This applies to your relations as to mine. Get interested in the case, not the person; and be cautious about this matter of too easy friendliness. It does harm, saps authority, and gives rise to needless disappointments.

I wish to say a few words as to the training—the self-training—in observation. You are much with the patient. Your reports will be of use in proportion to your watchfulness, and this must be constant and tactful, and not such as to annoy or call attention. You can not imagine how this quality grows by use. If a nurse sees what I do not or cannot or fail to see, and has the skill to write it clearly, that is the nurse I like to have. I think that I was not a good observer as a young man. I believe now that few things escape me. Really it is amazing how much value all sorts of trifles have in our work. I once had, as an illustration, a hysterical case seen to their despair by doctors all over the world. I asked her one day for cologne, of which usually a bottle stood on a varnished old table by her bedside. She said: "My maid upset it this morning on the table." I said: "Here?" "Yes." When I went out with the attendant M.D., I said: "This woman is drinking cologne. If it had been overturned on that table there would have been a white stain on the varnish. I have noticed that." My inference proved to be correct. Of what use, one would say beforehand, could it be in medicine to know that alcohol whitened varnish.

I come now to a very grave matter, one which is an almost daily source to me of exasperation. It is full of difficulties. And it is the conversation, or rather the talk, of nurses. The nurse just out of a great hospital is the worst sinner. If she does not chatter about her cases and operations and what she has seen, she is truly a delightful exception. Where and when nurses meet at table they are likely to do these things. Is it a fault of training? I do not know; but it is most annoying and most harmful. Do not discuss your patients with nurses or other patients. You do it either from love of gossip or because you lack resources—and here, I suspect, is the real trouble. You want education, or else you have no interest in books or things not personal.

Unless you can see and cure this, I must talk in vain; but the American woman has wonderful powers of self-education, and the nurse is wise who will try to profit by her occasional and long contacts with the refined and educated. Read, therefore, and try to think and take notes, and so become reasonable companions. If patients try to talk symptoms needlessly and seek to make you discuss what you have seen, plead the doctor's orders against this, and learn to turn aside such disastrous talk. All of you can become wiser as well as better, and no accomplishment in nursing is more excellent than to learn to read aloud well. No nurse can know too much or fail to find uses for accomplishments. I pleasantly recall a case in which two good nurses suffered defeat. A third won easily because she happened to sing well, and this entirely captured a patient none else could manage.

I cannot leave these criticisms without adding a word on manners. I have been told by doctors that they did not want ladies for nurses. I do. I know of no place where loveliness of manners, where good breeding, so help to win as in the sick-room. And this must be so because the best manners are children of both heart and head. Think, therefore, of this, and when women of gentle breeding and refinement study their ways and learn how much life is eased by knowing just how best and most agreeably to do the little things of every day existence. Now, modesty is a part of the best of manners, and here I know that some nurses fail over and over. I have heard gentlewomen say: "Yes, an admirable nurse, doctor, but not so entirely modest in all her ways as one would like."

As to cleanliness, I do not like to say a word. A nurse should bathe daily and contrive a screen, and remember what Florence Nightingale said—that one could be clean with a cupful of water and a little patience and desire to be clean. I like much to see a nurse in cap and apron. This neat white dress sets her apart, is a uniform and gives authority; and, too, I like it because the least spot shows. It is a sort of conscience for cleanliness, and quickly reports untidiness.

I think nothing more desirable than for a nurse to learn to control her emotions, no matter what may occur. Hospital experience is valuable in thus educating a woman. To have a nurse hysterical is to have her henceforth useless. To be surely ready and unmoved by unlooked-for emergencies is perhaps hardly to be acquired. It comes by nature. I once was in a steamboat collision in Holland. I saw at once six Dutch women in hysterical spasms, and one was a sister of charity. My American women remained undisturbed, largely because they were of a class taught always to repress all display of emotion; and it is the giving way to emotion which leads to much hysteria. Be careful, therefore.

Some nurses seem to me to plant the seed of opposition, and some carry with them the pleasant sunshine of peace.

There are nurses and nurses, and I would like to see some of them paid more largely than they are, and some I would like to see paid very little until they learned their business. I like to quote what I should have quoted before—the words of our dead surgeon, Agnew: "It is a great and good thing to feel that you are not always working for mere money."—*Dr. S. Weir Mitchell.*

